# Cognitive Changes and Brain Volume Reduction in Patients with Nonalcoholic Fatty Liver Disease

**DOI:** 10.1155/2018/9638797

**Published:** 2018-02-27

**Authors:** Branka Filipović, Olivera Marković, Vesna Đurić, Branislav Filipović

**Affiliations:** ^1^Faculty of Medicine, University of Belgrade, Dr Subotića Starijeg 8, Belgrade, Serbia; ^2^Department of Gastroenterohepatology, Clinical and Hospital Center Bežanijska Kosa, Autoput s/n, 11080 Belgrade, Serbia; ^3^Department of Hematology, Clinical and Hospital Center Bežanijska Kosa, Autoput s/n, 11080 Belgrade, Serbia; ^4^Clinical and Hospital Center “Dr Dragiša Mišović-Dedinje”, Belgrade, Serbia; ^5^Institute of Anatomy “Niko Miljanić”, Dr Subotića Starijeg 4/2, Belgrade, Serbia

## Abstract

Studies of psychological condition of patients suffering from nonalcoholic fatty liver disease are rather equivocal about the results: while some claim that NAFLD patients suffer from anxiety and depression more than non-NAFLD controls, others do not withstand those findings. Lower cognitive potentials have also been reported, both in patient related and in animal model-based investigations, and correlated with assessed brain tissue changes. We hypothesized that NAFLD, as a condition, affects the brain tissue and, subsequently, the cognitive state. So we compared findings in 40 NAFLD positive and 36 NAFLD negative patients and correlated their brain tissue volumes with the results of Montreal Cognitive Assessment (MoCA) test. Binomial logistic regression verified the influence of NAFLD state leading to lower cognitive potentials: odds ratio 0.096; 95% confidence interval (CI) 0.032–0.289; *p* < 0.001. Patients with NAFLD had a greater risk to suffer from the cognitive impairment and depression: RR = 3.9; 95% CI 1.815–8.381; *p* = 0.0005 and RR = 1.65; 95% CI 1.16–2.36; *p* = 0.006. NAFLD significantly influenced the cognitive deficit and tissue volume reduction and patients suffering from NAFLD had about four times higher risk of having a cognitive impairment.

## 1. Introduction

Psychological condition of patients with nonalcoholic fatty liver disease (NAFLD) still remains rather ambiguous. Studies related to the mentioned problem are dealing mostly with depression, but results obtained are equivocal: some studies confirmed the presence of depression in correlation with histological severity of NAFLD [1–4], while other publications denied the relationship between them [5, 6]. A systematic review by Macavei and coworkers (2016) [7] also emphasized the presence of depression and anxiety as the most frequent mood disturbances in NAFLD suffering patients. Petta and coworkers (2016) [8] outlined the relationship between decreased volume of brain white matter, lower cognitive potentials, and the type of fibrosis level in NAFLD. In both the entire cohort and NAFLD cases, only females older than 45 and with F2–F4 fibrosis level were independently associated with white matter lesions, mostly of mild grade. Some decrease in the blood oxygen concentration was revealed in female NAFLD patients and it was connected to lower cognitive performance in females affected by NAFLD [9].

The experimental investigation of NAFLD performed on Sprague-Dawley rats delineated the changes on hippocampal-related memory, but the deficit in hippocampal insulin signaling or brain-derived neurotrophic and insulin-like growth factor 1 appeared to have no influence on this [10].

We aimed to assess whether NAFLD influences the cognitive status in patients and if cognitive impairment correlates with lower volumes of various brain structures, including total brain white and gray matter volumes and volumes of the lateral ventricles in NAFLD patients.

## 2. Material and Methods

The study group involved 89 first diagnosed, therapy naive patients, with high level of aminotransferases, out of which 40 (22 men and 18 women) aged 34–57 (mean 47.88 ± 6.07) satisfied recruiting criteria, who were treated in the Department of Gastroenterology and Hepatology, or referred to the Gastroenterology outpatients service. Control group contained 30 patients, 16 men and 14 women, aged 39–53 (mean and standard deviation = 47.07 ± 6.68) who were referred to the outpatient service for other gastroenterological problems, mostly of the functional origin (functional dyspepsia and irritable bowel syndrome), that do not involve liver nor gall bladder and gall pathways pathology, which were excluded by the abdominal ultrasonography. The demographic data are presented on Table 1.

Recruiting criteria are as follows:

1. Older than 18
2. No previous history of viral hepatitis of any kind, haemochromatosis, autoimmune hepatitis, cirrhosis, or other chronic liver disease
3. No presence of severe cardiopulmonary disease
4. Absence of obstructive sleep apnea syndrome assessed by polysomnography
5. The absence of endocrinological disorders: hypothyroidism, hypercorticism, syndrome of the polycystic ovaria
6. No history or clinical signs of excessive alcohol abuse (>20 g/day for males and >10 g/day for females)
7. No psychiatric disease and/or psychiatric medication history or any other hepatotoxic drugs
8. No visible traces of the illicit drugs abuse: negative urine multiple drug test on 10 kinds of drugs: cannabinoids, opiates, amphetamines, 3,4-methylenedioxymethamphetamine, cocaine/crack, benzodiazepines, tricyclic antidepressants, barbiturates, methadone, and buprenorphine
9. No visible focal or diffuse changes in the gray matter of the brain on MRI
10. Fazekas score 0 on MRI scan: Fazekas score is the estimated level of the white matter vascular changes and is the aftermath of brain vessels atherosclerotic changes
11. Absence of any rheumatological disease
12. Patients who used antidiabetic drugs, insulin, antilipemic drugs, uricosuric drugs, steroids, and oral contraceptives were omitted from the study.

**Figure 1:**
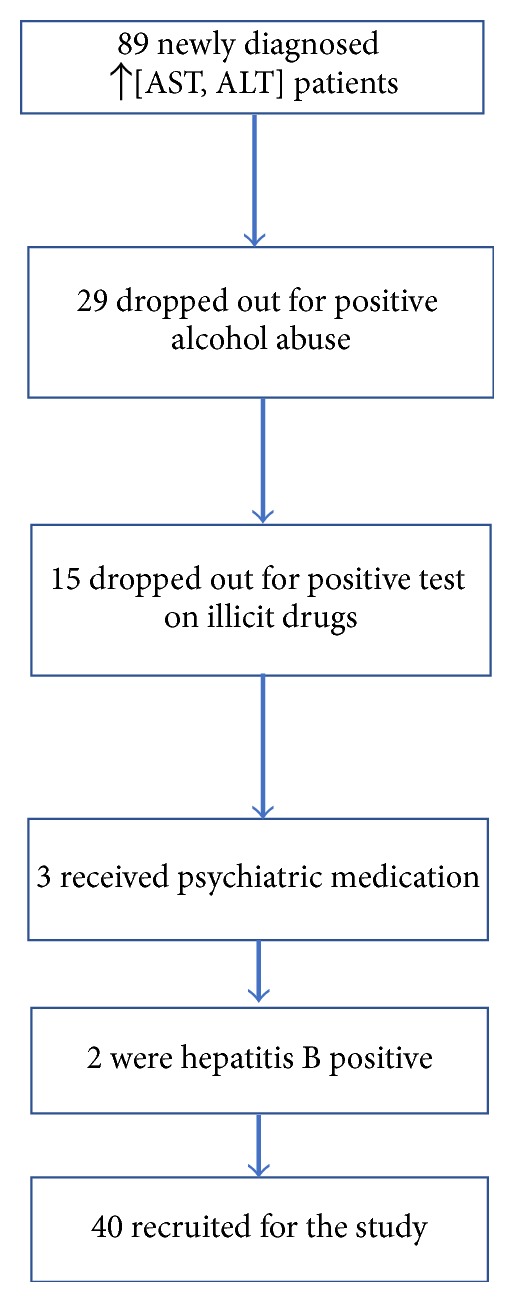
Flow diagram of patient selection from Department of Gastroenterohepatology.

Most of the patients who were dropped out fell on the alcohol (29) and drug abuse tests (15). Flow diagram is shown on Figure 1.

Upon admittance, all the patients underwent abdominal ultrasonography and MRI brain scanning.

Abdominal ultrasound was performed using a 3.5 MHz transducer, by an experienced gastroenterologist. Both subcostal and intercostal scannings were done. Images were captured in a standard fashion with the subject in the supine position and with the right arm raised above the head. In our clinical study we measured the following ultrasonographic parameters: anterior-posterior diameter of the liver (AP), homogeneity, echogenicity of the hepatic parenchyma, and the pancreas. Normal liver parenchyma was seen as solid homogenous echo texture, which was midway between the renal cortex and pancreatic echogenicity. Fatty liver disease was diagnosed as diffusely increased echogenicity of the hepatic parenchyma compared to the kidneys, vascular blurring, and deep-echo attenuation.

Sonographic evaluation (US) of hepatic steatosis was based on five criteria: parenchymal brightness, liver to kidney contrast, deep beam attenuation, bright vessel walls, and gallbladder wall definition [11, 12]. Diffuse hepatic steatosis was graded I to III: I, increased hepatic echogenicity with visible periportal and diaphragmatic echogenicity; II, increased hepatic echogenicity with imperceptible periportal echogenicity, without obscuration of the diaphragm; and III, increased hepatic echogenicity with imperceptible periportal echogenicity and obscuration of the diaphragm.

Blood samples were collected after 12 hours of fasting. Analyses included platelet count (PC), platelet indices (MPV, PCT, and PDW), liver function tests, aspartate aminotransferase (AST), alanine aminotransferase (ALT), gamma-glutamyl transferase (GGT), alkaline phosphatase (ALP), lipid profile including triglycerides, C-reactive protein (CRP), and fasting blood glycaemia and insulin levels, by using Ilab 650 (Instrumentation Laboratory, Milano, Italy).

Volume measurements of the gray and white matter and lateral ventricles of the brain were performed on 3D T1-weighted MR images (Phillips Inc., Holland); acquisition parameters were as follows: TR = 9.8 ms; TE = 4.6 ms; fl ip angle = 8; section thickness = 1.2 mm; number of sections = 120; no section gap; whole brain coverage; FOV = 224 mm; matrix = 192; reconstruction matrix = 256. Routine T2-weighted and FLAIR images were performed to rule out a mass lesion as a contributory factor to memory loss or cognitive decline. The structures were manually outlined and compared with automatic extraction of the regions of interest in commercially available software. The software finally computed the volumes required.

After the diagnostic procedures, all the subjects underwent psychological testing for cognitive impairments using Montreal Cognitive Assessment test, Serbian version [13]. Test has several levels of testing, alternating connections (connect Figure 1 with letter A and then A to 2, to B, etc.), visuoconstructive abilities (draw a cube and a clock in 11:10 position of clock hands), memory (numbers repeated in the same and reverse order), attention (tap whenever you hear a letter A), serial subtraction of 7, starting with a hundred (100 − 7 = 93, 93 − 7 = 86, etc.), sentence repeating, and verbal fluency. Maximal score is 30, 26 being the threshold for normal cognitive functioning.

Level of the depression was tested by Hamilton's depression rating scale of 21 items. Only first 17 items were scored: equal to or lower than 7, no depression; 8–13, mild depression; 14–18, moderate; 19–22, severe; equal to or higher than 23, very severe. For all the patients with depression diagnosis, a psychiatrist was consulted, and the subsequent therapy was individually prescribed. According to the protocol, patients were controlled every three months. The protocol involved liver examination by ultrasound, laboratory parameters, and psychiatric evaluation.

Statistical testing was performed by the commercially available software. Besides the measures of central tendency (mean and standard deviation (SD), minimum, and maximum), potential differences of mean values were assessed with one way analysis of variance (ANOVA) with Bonferroni post hoc correction, Student's *t*-test for independent samples for parametric, and chi square test for nonparametric data. Correlation was tested with Spearman and Pierson's correlation test. Possible dependence between the nonparametric parameters was estimated by binomial logistic regression. All the testing was performed on 95% probability level.

All the participants were acquainted in detail with the study aim and design before entering the program. They all signed a written consent afterwards.

## 3. Results

The cognitive status, according to MoCA index, was lower in NAFLD patients (Table 1). If we observe the cutoff value (26 points) and divide both groups into normal and subnormal groups, 26 patients in NAFLD group had lower MoCA score versus 6 control members (chi square = 19.23, df = 1, and *p* < 0,0001). Binomial logistic regression estimated significant influence of NAFLD on the reduced MoCA score: odds ratio 0.096, 95% confidence interval (CI) 0.032–0.289, and *p* < 0.001. Patients with NAFLD had a greater risk to suffer from cognitive impairment: RR = 3.9, 95% CI 1.815–8.381, and *p* = 0.0005, respectively.

Despite the fact that we did not evaluate differences in total brain, gray, and white volumes between the observed and control group, in NAFLD patients with lower MoCA score, the volumes of brain, gray, and white matter were significantly reduced: for brain volume, *t* = 2.71, df = 38, and *p* = 0.01, for gray matter volume, *t* = 2.71, df = 38, and *p* = 0.01 (absolutely correlated with brain volume), and for white matter, *t* = 2.31, df = 38, and *p* = 0.026. Furthermore, MoCA score values correlated with gray and white matter volumes, but not with total brain volume in NAFLD group. The increase in lateral ventricles volumes negatively correlated with total MoCA score (Table 2).

Difficulties appeared in NAFLD + group with questions that required postponed memory test to repeat the words after 5 minutes. Although no significant difference was obtained by chi square (chi = 3.5978, *p* = 0.0057), 22 examinees versus 12 controls did not solve the task. With question requiring attention, 28 examinees had more than two mistakes in tapping with pencil every time they heard the letter A, while the same mistake was revealed in controls in 9 cases (chi = 15.35, *p* = 0.00009).

Classified by Hamilton's depression score, there are more patients with moderate and severe depression in NAFLD group and this difference was verified by chi square test (Table 3). Furthermore, patients with NAFLD had a higher risk of havin depression: RR = 1.65, 95% CI 1.16–2.36, and *p* = 0.006.

Although persons with increased body mass index (BMI), hypertension, and metabolic syndrome dominated in the NAFLD positive group, neither BMI, hypertension, nor metabolic syndrome had influence on the cognitive status. HDL/LDL ratio was not significantly related to lower MoCA index, although the risk for atherosclerosis was expressed among examinees from NAFLD positive group. Neither transaminase level nor the ratio influenced significantly the cognitive deficit.

No gender based differences were evaluated in this study.

## 4. Discussion

This study represents authors' effort to evaluate the cognitive status of newly recruited NAFLD patients and to correlate it to the total brain volumes, volumes of gray and white matter, and volumes of the lateral ventricles. The major finding of our study is the assessment of higher number of persons with lower cognitive abilities in NAFLD patients. Higher risk for NAFLD patients to suffer from the cognitive impairment and independent influence of NAFLD on lower cognitive scores have also been evaluated. Previous investigations also correlated the cognitive deficit with white matter changes of vascular origin [8]. Some investigations outlined the increased connection between white matter changes in menopausal and postmenopausal females [14], but, as we had not obtained statistical correlation between age and gender with any kind of volumes of interest, we did not want to involve hormonal status in our investigation. Depleted cognitive performance has been noted in NAFLD patients: increased activity of alanine (ALT) and aspartate aminotransferase (AST) correlated with poorer performance on serial digit learning test and increased activity of ALT correlated with serial digit replacement test [15]. Serial digit learning test (SDLT) is a golden standard for functional magnetic resonance memory testing and consists of three independent series of nine (sometimes eight) single digits, in which examinee must repeat correctly after maximum 12 attempts, in five to ten minutes. The other test, serial digit replacement test (SDRT), is designed for attention testing and is based on the replacement of single digit by a prescheduled sign. Obviously, NAFLD patients have a problem with memory, also found in our study, but our patients manifested less problems in postponed memory test than in attention evaluation; moreover, we found no statistically relevant correlation between transaminases activity and cognition scores. On the other side, Petta and his coworkers (2016) [8] found no difference in cognitive state in NAFLD positive patients, with and without white matter lesions. For the mentioned evaluation, they used mini mental state questionnaire, applied mostly to estimate the presence of dementia. This is not a sensitive type of questionnaire and its application is not advisable for subtle cognitive changes, as manifested in NAFLD. Reduced gray and white matter volumes, obtained in our study, combined with the increased volumes of the lateral ventricles, may be used as an explanation why total brain volume did not differ between the examinees and controls, because controls had increased volumes of gray and white matter but lower volumes of the lateral ventricles. According to VanWagner et al. (2017) [16], total brain tissue volume did not differ in groups with and without NAFLD, while gray matter perfusion was lower in NAFLD patients.

Previous reports have outlined that higher levels of adiposity including BMI, waist circumference, subcutaneous adipose tissue, and volume of adipose tissue have all been associated with lower total brain volume [17, 18]. BMI, in particular, has been associated with regional brain gray matter volume decreases, although the location and magnitude of these decreases have been inconsistent [19–22]. Some investigations reported lower gray matter volume only in those with obesity (BMI ≥ 30 kg/m^2^) [18], although our investigation cannot confirm those findings. According to our results, BMI does not correlate with total brain, gray, or white matter volumes, nor with the depression grade.

Youssef et al. (2013) [3] published in their article that depression levels were associated with portal fibrosis grades (*p* = 0.038) and tended to be associated with hepatocyte ballooning grades (*p* = 0.085). In our study, 75% of NAFLD + examinees had depression versus 69% in cited publication [3]. Subclinical form of depression (e.g., mild + moderate) appeared in 28 out of 40 patients, while clinical form of depression (severe) was assessed in 2 patients. Our estimated risk for depression of 1.65 is related to overall NAFLD group, without difference for males and females. Gender differences in depression prevalence in NAFLD were outlined by Surdea-Blaga and Dumitraşcu (2011) [5], who stated that female NAFLD patients have higher relative risk for depression: 3.2 (95% CI = 1.6–6.7).

The previous investigation blamed insulin resistance for the predicted cognitive deficit in NAFLD + patients, indicating the prominent role of insulin resistance in Alzheimer's disease [23, 24]. Other studies, however, blamed the inflammatory process for cognitive impairment, relating it to the increased concentration of cytokines and adipokines, eventually associated with dysfunctional endothelium and oxidative stress, stating that it could be in the root of cognitive dysfunction in NAFLD patients [25–29]. Thickening of carotid arteries tunica intima, also reported in NAFLD patients and in patients with cognitive deficit as well, is the probable aftermath of the aforementioned process, which, as a definitive result, gives the volume reduction of gray and white matter and, consequently, cognitive impairment [30, 31]. Inflammatory mechanism should not be neglected as one of the causes of cognitive loss, but, according to our laboratory results, values of this inflammation marker were in the accepted range (0–10 mg/l) in both groups, without statistical difference (3.96 vs. 3.52 mg/l, examinees versus controls).

However, our study has some limitations:

1. This is a referral, not cohort study, restricted only to the patients referred to our department and outpatient clinics.
2. It is limited to newly diagnosed, therapy naive patients.
3. Only noninvasive tests were used for the NAFLD estimation.
4. The diagnosis is dependable on ultrasonographist skills and experience.

## 5. Conclusion

According to our results, cognitive deficit, assessed by Montreal Cognitive Assessment test, is more pronounced in persons with NAFLD. NAFLD significantly influenced the cognitive deficit and patients suffering from NAFLD had about four times higher risk to have a cognitive impairment. Depleted MoCA score correlated with the white and gray matter volume reduction. NAFLD patients are at higher risk to suffer from depression, which is, most probably, related to the revealed volume reductions as well. Nonetheless, deficit is not in a verified correlation with BMI, found to be higher in the examined group, nor with the activities of liver transaminases, although the higher concentration of AST was revealed in NAFLD patients. Future investigations should be conducted to elucidate still insufficiently known exact mechanism of cognitive deficit pathogenesis, and the therapy of NAFLD is advisable to act in such a way as to prevent it.

## Figures and Tables

**Table 1:** Demographic characteristics of examined cohort.

	Examined (40)		Control (36)		Difference and significance	
Age (yrs., mean ± SD)	47.88 ± 6.07		47.07 ± 6.68		NS	
Sex (male/female)	22/18		16/14		NS	
Body mass index (BMI, kg/m^2^, mean ± SD)	32.04 ± 6.67		22.18 ± 4.43		*p* < 0.001	
Diabetes type 2 (yes/no)	12/28		9/27		NS	
Hypertension (yes/no)	32/8		16/20		*p* < 0.0001 Spearman correlation NAFLD + and hypertension, *p* < 0.0001	
HDL/LDL ratio (mean ± SE)	4.49 ± 0.23		2.94 ± 0.18		*p* < 0.0001	
Status according to BMI:						
Normal	5		21		*p* = 0.000028	
Overweight	15		11			
Obese	20		4			
Metabolic syndrome (yes/no)	34/6		8/28		Chi square, *p* < 0.00001	
MoCA score (mean ± SD)	24.07 ± 3.18		27.17 ± 2.35		*p* < 0.001	
Total brain volume (cm^3^, mean ± SD)	1388 ± 127.98		1417 ± 191.83		NS	
Gray matter volume (gmw in cm^3^ mean ± SD)	405 ± 37.42		414 ± 56.09		NS	
White matter volume (wmw, in cm^3^ mean ± SD)	338 ± 31.21		344 ± 46.83		NS	
Lateral ventricle volume, right/left (lvlr, lvll, in cm^3^ mean ± SD)	5.97 ± 0.61	6.07 ± 0.62	5.64 ± 0.37	5.63 ± 0.37	*p* = 0.009^*∗*^	*p* < 0.001^*∗*^
AST (U/l, mean ± SE)	39.97 ± 2.61		24.03 ±3.26		*p* < 0.001	
ALT (U/l mean ± SE)	39.11 ± 2.86		36.71 ± 3.5		NS	
AST/ALT ratio (mean ± SE)	1.13 ± 0.11		0.7 ± 0.06		*p* = 0.01	
C-reactive protein (mg/l, mean ± SE)	3.96 ± 0.45		3.52 ± 0.51		NS	
Fasting glycaemia (mmol/l, l mean ± SE)	5.8 ± 0.19		3.71 ± 0.25		*p* < 0.001	
Triglycerides (mmol/l, l mean ± SE)	2.87 ± 0.18		1.71 ± 0.17		*p* < 0.001	

NS, not significant; SE, standard error. ^*∗*^Lateral ventricle volumes.

**Table 2:** Pierson's correlation of MoCA score values and observed volumes.

Volume	*B*	Beta	Significance
Total brain volume	0.025	1.5	NS
latvl	−39.18	−7.48	*p* = 0.005
latvr	−38.01	−7.46	*p* = 0.007
gmw	0.019	0.279	*p* = 0.015
wmw	0.2	2.89	*p* = 0.009

Latvl: lateral ventricle volume, left hemisphere; latvr: lateral ventricle volume, right hemisphere; gmw: gray matter volume; wmw: white matter volume.

**Table 3:** Depression grades in observed and control groups.

	Hamilton severity^*∗*^				Total
	No depression	Mild	Moderate	Severe	
Group					
NAFLD+	7	17	14	2	40
NAFLD−	18	15	3	0	36
Total	25	32	17	2	76

^*∗*^Chi square = 13.91, df = 3, and *p* < 0,0001.
